# Anti-Amyloid Therapies for Alzheimer’s Disease: Progress, Pitfalls, and the Path Ahead

**DOI:** 10.3390/ijms26199529

**Published:** 2025-09-29

**Authors:** Vasileios Papaliagkas

**Affiliations:** Laboratory of Neurophysiology and Cognitive Neuroscience, Department of Biomedical Sciences, School of Health Sciences, International Hellenic University, 57001 Thessaloniki, Greece; vpapaliagkas@ihu.gr

**Keywords:** Alzheimer’s disease, lecanemab, donanemab, aducanumab, amyloid, ARIA, APOE, disease-modifying therapy, biomarkers, value-based care

## Abstract

Anti-amyloid monoclonal antibodies have finally achieved their translational breakthrough after many years of unmet expectations. The FDA granted traditional approval to lecanemab in July 2023, and the European Medicines Agency approved it in late 2024 with specific genetic restrictions; meanwhile, donanemab received FDA approval in July 2024 and EMA marketing authorization just one month ago. These agents consistently clear cerebral amyloid and slow clinical decline modestly in early-stage, biomarker-confirmed Alzheimer’s disease (AD). On the other hand, they also create significant safety risks, including amyloid-related imaging abnormalities (ARIA) and substantial operational requirements for health systems that are already under pressure. Therefore, precise risk management based on APOE genotyping and the presence of cerebral amyloid angiopathy and cerebral microbleeds should be performed before therapy is initiated. The near-term agenda should prioritize the following areas of study: (1) biomarker-driven front-end triage (including emerging plasma assays); (2) ARIA-aware care pathways and shared decision making; (3) outcome-based coverage and rational pricing; (4) clinical trials that layer anti-amyloid therapy into combinatorial strategies targeting tau protein, neuroinflammation, and synaptic resilience.

## 1. Introduction

The amyloid cascade hypothesis has been the leading theory in Alzheimer’s disease (AD) drug development for the past thirty years. Plaque reduction has been successful in laboratory settings, yet clinical translation has failed repeatedly until recently. The phase 3 CLARITY-AD trial of lecanemab [1], and the phase 3 TRAILBLAZER-ALZ 2 trial of donanemab [2] showed statistically significant slowing of cognitive and functional decline in early AD alongside substantial amyloid clearance. Lecanemab subsequently gained traditional FDA approval on 6 July 2023 and later a positive CHMP opinion (November 2024) leading to EU authorization with genotype-related restrictions. Donanemab was approved by the FDA on 2 July 2024, and on 25 July 2025, it was granted marketing authorization by the EMA [3]. The first antibody to gain accelerated approval in the U.S. market in 2021 was aducanumab, which Biogen discontinued in 2024, thus ending a disputed period and making way for agents with better confirmatory evidence [4]. Anti-amyloid monoclonal antibodies represent a significant advancement in the treatment of early Alzheimer’s disease. This opinion article aims to provide a perspective on these antibodies with a focus on clinical trial results, patient selection, ARIA risk management, shared decision making, and strategies for optimizing treatment outcomes in resource-constrained settings.

## 2. Results

### 2.1. Clinical Trial Outcomes

The CLARITY-AD study is a Phase 3 18-month, double-blind, placebo-controlled study that demonstrated lecanemab’s ability to treat early AD patients by reducing CDR-SB scores and other measures of cognition (i.e., ADAS-cog, ADCOMS, ADCS-MCI-ADL) as well as achieving substantial amyloid burden reduction on PET (measured in centiloids). In particular, the adjusted mean change from baseline at 18 months in the CDR-SB score was 1.21 in the lecanemab group and 1.66 in the placebo group (difference, −0.45; 95% CI, −0.67 to −0.23; *p* < 0.001); for amyloid burden, the adjusted mean change was −55.48 centiloids in the lecanemab group and 3.64 centiloids in the placebo group (difference, −59.12 centiloids; 95% CI, −62.64 to −55.60; *p* < 0.001). The amyloid positivity cutoff was 30 centiloids [1]. TRAILBLAZER-ALZ 2 is a Phase 3, 76-week, double-blind, placebo-controlled study that showed that donanemab reduced clinical progression in patients with early symptomatic AD who had amyloid and tau pathology, while using a treatment cessation protocol after amyloid clearance. The amyloid plaque level decreased by 88.0 Centiloids (95% CI, −90.20 to −85.87) with the donanemab treatment and increased by 0.2 Centiloids (95% CI, −1.91 to 2.26) in the placebo group in the low-/medium-tau population. Amyloid positivity cutoff was 37 centiloids [2]. The clinical trials’ characteristics are presented in Table 1.

### 2.2. Safety and Risk Management

The major adverse events associated with anti-amyloid therapy are amyloid-related imaging abnormalities (ARIA) that include edema or effusion (ARIA-E) and cerebral micro/macrohemorrhage or siderosis (ARIA-H).

#### 2.2.1. ARIA-E (Edema/Effusion)

ARIA-E is characterized as vasogenic edema and sulcal effusions best seen on fluid-attenuated inversion recovery (FLAIR) MRI sequences. The blood–brain barrier experiences brief disruptions during amyloid clearance which leads to this condition.

Most ARIA-E cases present without symptoms. When symptoms occur, they are usually mild and include headaches, confusion, visual disturbances, nausea, and vomiting. The extent and severity of edema/effusion on MRI determines the grading of ARIA-E through a standardized system, such as the 3- and 5-point ARIA-E severity scales (SSAE-3/-5) that are used in most AD trials [5].

#### 2.2.2. Management

Management of asymptomatic ARIA-E involves careful monitoring for symptoms and monthly non-contrast MRI scans until ARIA-E has resolved. In moderate–severe radiographic ARIA-E cases or symptomatic patients, therapy should be held until ARIA-E or any clinical symptoms are resolved. If symptoms persist, patients should be treated with corticosteroids (eg, 1 g prednisone for 3 to 5 days followed by a several-week taper) [6].

#### 2.2.3. ARIA-H (Hemorrhage/Hemosiderosis)

ARIA-H is characterized by microhemorrhages and superficial siderosis with iron deposition, that are more visible in Gradient Echo (GRE) or susceptibility-weighted imaging (SWI) MRI sequences. Most ARIA-H cases are asymptomatic. ARIA-H symptoms that become noticeable in patients lead to focal neurological problems, seizures, and cognitive deterioration. The grading system for ARIA-H depends on the number and size of microhemorrhages and the extent of superficial siderosis.

#### 2.2.4. Management

The treatment approach for asymptomatic ARIA-H is continuing treatment with close monitoring, whereas temporary suspension of the anti-amyloid therapy is often recommended for more severe cases or if symptoms worsen.

#### 2.2.5. Termination of Therapy

Anti-amyloid therapy should be stopped in the following circumstances: (a) severe cases; (b) when ≥2 episodes of ARIA occur, regardless of clinical or radiologic severity; (c) when more than 10 microhemorrhages occur since the initiation of treatment; (d) any condition that requires treatment with anticoagulants [6].

The administration of anticoagulants (warfarin, hepatin, and DOACs) and thrombolytic agents (tPA) to patients undergoing anti-amyloid therapy needs proper evaluation because of elevated ARIA-H risks.

#### 2.2.6. Anticoagulation

Due to the increased risk for macrohemorrhages associated with ARIA, appropriate use recommendations (AURs) advise against the use of lecanemab and donanemab in patients taking anticoagulants until additional safety data are available [6,7].

#### 2.2.7. Thrombolysis

Administration of tPA for acute ischemic stroke treatment in patients taking anti-amyloid medications poses a high risk of bleeding complications. Fatal multifocal brain hemorrhages were reported in one patient treated with lecanemab [8] and one patient treated with donanemab [9]. As a result, AUR for lecanemab and donanemab do not recommend the use of thrombolytics [6,7] The current American heart association guidelines advise that healthcare providers should exercise extreme caution when treating patients with thrombolysis because they need to evaluate all potential risks and advantages. Factors that should be taken into consideration as risk factors for ARIA are the following: recency of immunotherapy initiation, APOE genotype, and baseline CAA markers [10].

**Figure 1:**
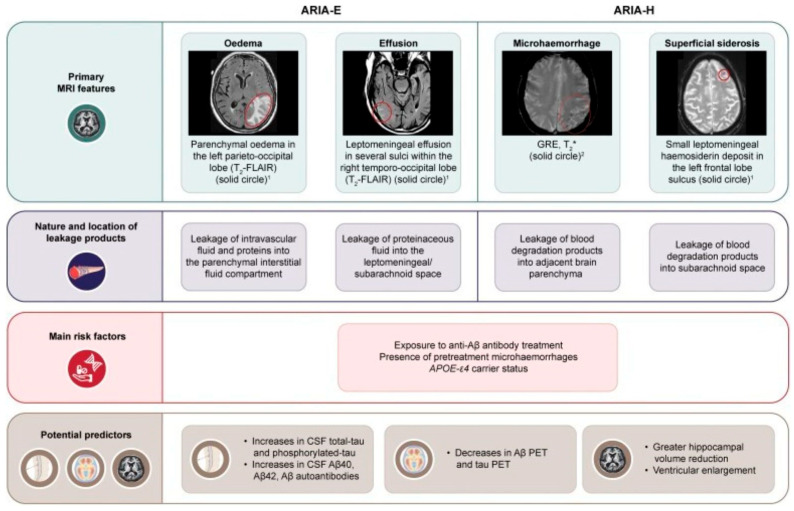
Characteristics of ARIA-H and ARIA-H, that include primary MRI features, nature, location, main risk factors, and potential predictors. Figure by Hampel et al. [14], licensed under CC BY 4.0.

The CLARITY-AD study showed that ARIA-E developed in 12.6% of lecanemab-treated patients and ARIA-H appeared in 17.3% of lecanemab-treated patients. APOE ε4 homozygotes had more severe and frequent radiographic changes, with ARIA incidence increasing to 32.6%. The ARIA-E burden of donanemab appears to be comparable to or possibly greater than that of aducanumab. The risk is again increased in ε4 homozygotes to 40.6%. Moreover, the presence of preexisting cerebral microhemorrhages increases the risk of ARIA-H. These risks necessitate frequent MRI monitoring and careful management of anticoagulation and thrombolysis decisions [11,12,13]. The main characteristics of ARIA, including the main risk factors and predictors, are presented in Figure 1 [14].

### 2.3. Patient and Clinical Decision Making

The general slowing rate of AD is moderate, but patients experience different rates of progression. Preservation of independence stands as a crucial factor for some, whereas others consider the adverse reactions to be more significant than the potential benefits. It is mandatory that patients are informed about the potential modest benefits of the anti-amyloid therapies versus possible risks. Due to the diverse characteristics of AD patients, it is important to implement shared decision making; this must be the core ethical principle of prescribing processes, as it takes into consideration a patient’s personal goals, priorities, and comorbidities as well as their genotypes and caregiver capacities [15].

### 2.4. Regulatory Divergence and Real-World Access

The FDA’s traditional approvals reflect confidence that amyloid lowering with correlated clinical benefit constitutes disease modification in early AD. EMA approved lecanemab (2024) with particular use conditions but rejected donanemab (2025) because of an unfavorable benefit–risk assessment in its application, before marketing authorization, about one month ago. This transatlantic difference reveals actual doubts about balancing clinical effects for groups against safety indicators and operational challenges [16,17].

The Centers for Medicare & Medicaid Services (CMS) offers Coverage with Evidence Development (CED) for FDA-approved anti-amyloid antibodies. The policy demands that Medicare patients join approved registries that track real-world treatment results. The requirement for registry participation restricts treatment access for patients who cannot reach participating centers or fulfill data collection obligations. The first CED policy for aducanumab imposed PET scan requirements, which created unequal treatment access for rural patients because PET imaging services are scarce in these areas. The current policy change has not resolved the problem of limited access to specialized neurology care and infusion centers, which affect numerous patients. The policy fails to resolve the issues of high patient expenses for medical costs, transportation fees, and work absences, which intensify the existing health inequalities between different social classes.

Future policies need to establish active solutions, which include expanding mobile PET scanner availability, offering financial help for transportation, and adding patient feedback to registry systems. The practical result is that coverage exists, but clinics must offer infusions, brain MRI procedure series, and neurology and biomarker workflows to meet label and safety requirements [18].

### 2.5. Treatment Costs

Eisai established the annual price of lecanemab to be USD 26,500 [19] while Lilly set that of donanemab at approximately USD 32,000 for a standard 12-month treatment period [20], without considering diagnostic and monitoring expenses (e.g., PET/MRI, genomics, laboratory). Independent assessors such as ICER have questioned the drug value at these financial costs [21].

### 2.6. Risk Management and Precision Medicine

Two developments are making anti-amyloid therapy safer and more patient-centered right now, as described below.

(a) Risk factors for ARIA: APOE ε4 homozygosity substantially elevates ARIA risk with both lecanemab and donanemab. Contemporary appropriate use recommendations (AURs) therefore advocate APOE genotyping to support risk discussions and triage, avoidance of anticoagulants/thrombolytics, and frequent MRI monitoring [6,7]. A comprehensive risk assessment should also consider other clinical and imaging markers:

- Patients with findings suggestive of cerebral amyloid angiopathy on brain MRI, such as the presence of cerebral microhemorrhages (CMH) and cortical superficial siderosis, should be considered to be at a higher risk for ARIA-H and may require more frequent monitoring.
- The presence of CMH should be taken into consideration, as patients with multiple baseline CMHs, particularly those located in cortical or subcortical regions, are at higher risk of ARIA.

(b) Structured care pathways: The current stroke/vascular neurology guidance includes specific recommendations for thrombolysis in patients who receive anti-amyloid therapy because ARIA can present in a similar way with ischemic stroke and thrombolytics may cause hemorrhage. Embedding these guardrails into emergency protocols is essential for real-world use scales [10]. Donanemab’s “finite dosing” (with planned discontinuation once amyloid clears) is attractive both clinically and economically, if clearance translates to durable benefit without rebound risk. This hypothesis requires future validation in regular practice and extended follow-up.

### 2.7. Initial Screening

Biomarkers are considered to be the new gatekeeper. The success of anti-amyloid therapy depends on the ability to find cases at a large scale. PET and CSF remain gold standards for confirming amyloid pathology, but widespread access is limited. The availability of plasma phosphorylated tau (p-tau) assays has increased since May 2025, when Lumipulse p-tau217 received FDA clearance as the first in vitro diagnostic test [22,23]. This development allows for affordable pre-screening and testing prioritization to confirm results. Health systems need to expedite the validation process for these assays in order to integrate them into diagnostic pathways, but it should be understood that they currently function as additional and supportive tools rather than definite diagnostic tests.

### 2.8. Health System Implementation Challenges

The implementation of coverage has not reached projected levels, because of operational challenges, which include limited access to both infusion sites and MRIs for baseline and serial monitoring. In addition, there is a lack of neurologists and neuroradiologists who are familiar with the identification and management of ARIAs. Essential diagnostic tests such as amyloid PET and particularly tau PET are not available in all countries. The early U.S. experience with lecanemab demonstrates this tension and shows that health system readiness rather than regulatory status determines access. The success of these treatments will depend on equal investments in nurse-led infusion programs, standardized MRI protocols, and centralized registries [24].

The average benefit levels together with high delivery expenses create challenges for achieving traditional, cost-effective standards. The analyses conducted by ICER demonstrate that value-consistent pricing for lecanemab should be set at levels lower than its current list price, thus supporting this approach [21]. ARIA risk, MRI access, and specialty care shortages disproportionately affect rural and minoritized communities. The introduction of disease-modifying therapy through mobile MRI and travel support and decentralized monitoring systems could potentially increase health disparities instead of reducing them. The preservation of any function for patients with progressive neurodegenerative illnesses becomes valuable during their remaining few months of life.

## 3. Future Directions

Three main priorities stand out in the case of anti-amyloid treatment in AD:

1. Combination and sequence strategies: The combination of anti-amyloid therapies with anti-tau agents, microglial modulators, neuroprotective synaptic agents, and lifestyle/digital therapies in trials may enhance benefits, particularly when they are initiated before symptom onset in individuals at high risk.
2. Adaptive dosing and de-escalation: The current dosing schedules for anti-amyloid treatments follow established protocols that are derived from clinical trial outcomes. Neuroscientists are now exploring adaptive dosing methods, which adjust treatment amounts and administration schedules according to patient-specific factors and treatment outcomes. These methods enable better treatment results, while minimizing ARIA risks and producing cost-effective outcomes. In particular, titration schemes might reduce ARIA incidence in ε4 homozygotes. The finite-course concept of donanemab enables us to study the potential safety of extending maintenance intervals after clearance of the drug [25].
3. Learning health systems: The U.S. Medicare registry requirement functions as a supportive structure, which enables the development of pragmatic evidence through safety signals (e.g., post-thrombolysis hemorrhage), equity metrics, and patient-reported outcomes that help improve AURs and payer policies [18]. The complete potential of learning health systems in this field depends on solving data quality problems and privacy issues, as well as achieving interoperability and addressing resource limitations.

## 4. Conclusions

Dementia researchers have achieved a historic goal, the development of disease-modifying therapies which remove amyloid deposits and reduce the progression of early AD. However, this achieved progress remains currently unstable worldwide, because it requires early diagnosis, multifactorial risk control, and continuous MRI surveillance as well as strong health system capabilities which numerous areas do not possess. The industry should welcome regulatory and payer scrutiny of value because it needs to develop safer dosing and simpler care models and clinicians should maintain their right to shared decision making. The author suggests that the ethical path forward is not to oversell or dismiss these agents, but to use them wisely while building the next generation—combinations, pre-symptomatic interventions, and targets beyond amyloid—that could convert today’s modest slowing into a more effective management of dementia.

## Figures and Tables

**Table 1:** Characteristics of clinical trials.

	Clarity-Ad (Lecanemab)	Trailblazer-Alz 2 (Donanemab)
Study Design	Phase 3, double-blind, placebo-controlled	Phase 3, double-blind, placebo-controlled
Inclusion Criteria	Patients with MCI due to AD or mild AD dementia; Global CDR 0.5 or 1.0 and CDR Memory Box score > 0.5; MMSE score ≥ 22 and ≤30; WMS-IV LMII ≥ 1 SD below age-adjusted mean; Amyloid pathology confirmed; Aged 50 to 90 years.	Patients with early symptomatic Alzheimer disease (mild cognitive impairment [MCI] or Alzheimer disease with mild dementia; MMSE score 20–28; Amyloid pathology (≥37 Centiloids) assessed with 18F-florbetapir or 18F-florbetabenpositron emission tomography (PET); Presence of tau pathology assessed by 18F-flortaucipir PET imaging with central image evaluation.
N(Participants)	N = 1759 (859 patients, 875 placebo)	N = 1736 (860 patients, 878 placebo)
Treatment Duration	18 months	76 weeks
Dosage	Lecanemab 10 mg/kg every 2 weeks	Donanemab (700 mg for the first 3 doses and 1400 mg thereafter) every 4 weeks
Efficacy Outcomes		
Primary Endpoint	The change in the score on the Clinical Dementia Rating–Sum of Boxes (CDR-SB)18 from baseline at 18 months.	The change in the iADRS score from baseline to 76 weeks in either the low-/medium-tau population or combined (low/medium and high tau) population.
Change in Primary Endpoint	1.21 in the lecanemab group and 1.66 in the placebo group (difference, −0.45; 95% confidence interval [CI], −0.67 to −0.23; *p* < 0.001)	−6.02 (95% CI, −7.01 to −5.03) in the donanemab group and −9.27 (95% CI, −10.23 to −8.31) in the placebo group (difference, 3.25 [95% CI, 1.88–4.62]; *p* < 0.001), representing a 35.1% (95% CI, 19.90–50.23%) slowing of disease progression
Key Secondary Endpoints	Mean differences between the two groups in the change from baseline favoring lecanemab were as follows: Adjusted mean change from baseline at 18 months was as follows: −55.48 centiloids in the lecanemab group and 3.64 centiloids in the placebo group (difference, −59.12 centiloids; 95% CI, −62.64 to −55.60; *p* < 0.001); For the ADAS-cog14 score, −1.44 (95% CI, −2.27 to −0.61; *p* < 0.001); For the ADCOMS, −0.050 (95% CI, −0.074 to −0.027; *p* < 0.001); and for the ADCS-MCI-ADL score, 2.0 (95% CI, 1.2 to 2.8; *p* < 0.001).	Least squares mean changes were as follows: −0.67 (95% CI, −0.95 to −0.40) (36.0% [95% CI, 20.76–51.15%] slowing of clinical progression) for CDR-SB; 1.83 (95% CI, 0.91–2.75) (39.9% [95% CI, 19.15–60.58%] slowing of clinical progression) for ADCS-iADL; −1.52 (95% CI, −2.25 to −0.79) (32.4% [95% CI, 16.55–48.35%] slowing of clinical progression) for ADAS-Cog.
Safety Data		
ARIA-E Incidence APOE e4 carrier	12.6% with lecanemab,1.7% with placebo. Heterozygote 10.9% Homozygote 32.6%	24.0% with donanemab, 2.1% with placebo Heterozygote 22.8% Homozygote 40.6%
ARIA-H Incidence APOE e4 carrier	17.3% with lecanemab, 9.0% with placebo Heterozygote 14% Homozygote 39%	31.4% with donanemab, 13.6% with placebo Heterozygote 32.3% Homozygote 50.3%
Serious Adverse Events	14.0% with lecanemab, 11.3% with placebo	17.4% with donanemab, 15.8% with placebo

## Data Availability

Not applicable.
